# A critical appraisal of guidelines used for management of severe acute malnutrition in South Africa’s referral system

**DOI:** 10.1186/s12961-017-0255-z

**Published:** 2017-10-18

**Authors:** Faith Nankasa Mambulu-Chikankheni, John Eyles, Ejemai Amaize Eboreime, Prudence Ditlopo

**Affiliations:** 1 Centre for Health Policy, School of Public Health, University of the Witwatersrand, Johannesburg, South Africa; 2 School of Geography and Earth Sciences, McMaster University, Hamilton, Canada; 3 Department of Planning, Research and Statistics, National Primary Healthcare Development Agency, Abuja, Nigeria

**Keywords:** Severe acute malnutrition, Referral healthcare, South Africa, Guideline

## Abstract

**Background:**

Focusing on healthcare referral processes for children with severe acute malnutrition (SAM) in South Africa, this paper discusses the comprehensiveness of documents (global and national) that guide the country’s SAM healthcare. This research is relevant because South African studies on SAM mostly examine the implementation of WHO guidelines in hospitals, making their technical relevance to the country’s lower level and referral healthcare system under-explored.

**Methods:**

To add to both literature and methods for studying SAM healthcare, we critically appraised four child healthcare guidelines (global and national) and conducted complementary expert interviews (*n* = 5). Combining both methods enabled us to examine the comprehensiveness of the documents as related to guiding SAM healthcare within the country’s referral system as well as the credibility (rigour and stakeholder representation) of the guideline documents’ development process.

**Results:**

None of the guidelines appraised covered all steps of SAM referrals; however, each addressed certain steps thoroughly, apart from transit care. Our study also revealed that national documents were mostly modelled after WHO guidelines but were not explicitly adapted to local context. Furthermore, we found most guidelines’ formulation processes to be unclear and stakeholder involvement in the process to be minimal.

**Conclusion:**

In adapting guidelines for management of SAM in South Africa, it is important that local context applicability is taken into consideration. In doing this, wider stakeholder involvement is essential; this is important because factors that affect SAM management go beyond in-hospital care. Community, civil society, medical and administrative involvement during guideline formulation processes will enhance acceptability and adherence to the guidelines.

## Background

Malnutrition accounts for 50% to 60% of hospital child deaths in sub-Saharan Africa [1]. Out of all of South Africa’s under-five deaths audited between 2012 and 2013, 30% were associated with severe acute malnutrition (SAM), 29% with under-weight for age, 1% with over-weight for age, 34% with normal nutrition status and 6% were unknown [2]. Children with SAM have a higher risk of death, estimated at 5–20 times above that of children with normal nutrition status since the condition’s complications (hypoglycaemia, hypothermia, dehydration, etc.) are deadly within 48 hours if not stabilised [3]. SAM is confirmed when children aged 6–59 months have a height-for-weight or length of less than 3 standard deviations or a z-score of less than –3, bilateral pitting pedal oedema and less than 115 mm upper-arm-circumference during a malnutrition diagnosis [3, 4]. Hospital SAM-related deaths result from inappropriate case management at facility level during primary healthcare referral processes and follow-up after discharge [3]. It is therefore important to understand the in-hospital SAM care by exploring the facets that may contribute to the mismanagement of these cases within the entire healthcare services.

As noted by South Africa’s national department of health [3], the referral process is vital in ensuring the reduction of SAM deaths (fatality rates) within the healthcare services. A few studies that have examined paediatric and other healthcare referrals in South Africa have not necessarily focused on SAM; however, they indicate non-adherence to referral protocols and clinical guidelines for healthcare by providers and non-compliance to referrals by consumers [5–7]. Therefore, there is a need for a critical analysis of the protocols for healthcare providers in the context of managing and referring SAM cases, i.e. how does non-compliance manifest itself? While a number of scholars have attempted to explore in-hospital SAM mortality by measuring the quality of in-hospital care through an assessment of the applicability of 10 WHO guidelines for malnutrition management (a global 10-step guide for managing and rehabilitating SAM cases at hospital level), guideline quality is barely analysed [1]. There is also a need for a comprehensive understanding of the guidelines discussing the decision to refer and of care in preparation for, during and when receiving SAM referrals.

This paper aims to understand the complexity of guidelines for care and referral processes of SAM in South Africa. Specifically, we (1) identified guidelines related to SAM care and referral processes (from examination, decision to refer, care involved in transit and at receiving facility and during back referrals); (2) examined the quality of guidelines that are used in South Africa and (3) discussed the implications of the strengths and shortfalls of the documents reviewed.

### A critical review of studies on SAM healthcare policies and guidelines

Healthcare is best performed with the use of policy guidelines or protocols. Waterlow [8] found that detailed and minimal errors in malnutrition treatment were associated with the use of standardised protocols or guidelines. Guidelines in clinical healthcare practice are developed systematically to aid practitioners when deciding relevant healthcare treatments and pathways for specific clinical conditions [9]. The clinical practice guidelines are meant to reduce variations in handling specific ailments, thus regarded as rigid support tools by some scholars [10]. However, evidence-based guidelines are known to improve patient care and outcomes as well as promote practitioners’ positive attitudes and efficient use of facility resources [11]. It is therefore vital to explore whether guidelines for healthcare meet this technical quality. In South Africa, global (WHO based), national-based or local guidelines contain information on SAM management; however, they are not equally researched. Specifically, most South African studies examine global guidelines, including the integrated management of childhood illnesses (IMCI) and WHO’s Guidelines for the Inpatient Treatment of Severely Malnourished Children (WHO-Steps), rather than the national or local guidelines for managing or referring SAM [1, 12–14]. Therefore, this critical review will enable a discussion of the quality of all relevant SAM guidelines.

South Africa-based studies that have previously explored SAM treatment guidelines have only focused on adaptation of WHO-Steps for in-patient care of children with SAM to rural hospitals. Such studies acknowledge the feasibility of implementing WHO-Steps of managing SAM in rural hospital settings. However, omission of some procedures (i.e. feeding per every 3 hours at night and giving routine antibiotics) by health workers was a hindrance to the guidelines’ full implementation [13, 14]. To resolve such problems, in-service training, support and supervision are mostly recommended, but focus is seldom vested on whether the WHO guidelines were rigorously developed to suit the implementing contexts [1]. On the contrary, Deen et al.’s [13] study in 12 African hospitals found that clinical staff raised concerns about the evidence of using the same antibiotic regimen for children with marasmus and kwashiorkor, thus leading to the guidelines being implemented differently in various contexts. As a result of the scepticism over antibiotic recommendations in guideline documents, some hospitals gave antibiotics to all children with malnutrition while others only administered them to children with SAM [13]. Applicability of guidelines meant for global use may be difficult to achieve in low- and middle-income countries despite extensive fieldwork, involvement of SAM expert teams and revisions at development stage [13]. In consideration of the scepticism over the credibility of guidelines and a need for contextual-based SAM healthcare protocols, there is a need to assess SAM guideline development rigour and contextualisation.

Also neglected in studies that explore guidelines on SAM healthcare is the assessment of whether the documents are clear on practice, up to date (with revision plan) and if they were developed rigorously, by whom and under whose influence. In a study meant to appraise WHO guidelines for maternal health, Polus et al. [15] acknowledged WHO’s vital role in provision of global guidance to healthcare. It is therefore apt to explore SAM guidelines on both a technical level and a management, dissemination and implementation one. Additionally, since most studies on implementation of SAM guidelines are conducted at hospital level, focusing on how relevant they are to primary healthcare (PHC; composed of clinics and community health centres) and during referrals to other levels of care is an analytic gap.

South Africa did not have a national framework for managing malnutrition until 2012; prior to that, four of nine provinces adopted the WHO-Steps guidelines. The rest of the provinces utilised IMCI and South Africa’s standard treatment guidelines and essential drugs list for hospital level paediatrics, which were not explicitly explored in the context of SAM [3]. By focusing on SAM aspects of the guidelines currently implemented and how specific they are to context as well as how translatable they are to clinical operations implied in a referral framework, this analysis will utilise a specific technique, namely the Appraisal of Guidelines for Research and Evaluation (AGREE) tool, as well as WHO components of the referral process.

## Methods

### Study design

**Fig. 1:**
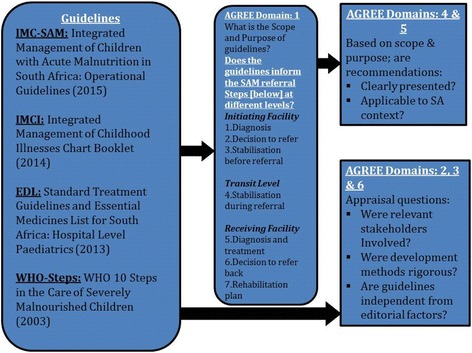
Integrated analytical framework for appraising SAM guidelines

This study is a critical appraisal of care guidelines for SAM care that inform the referral process in South Africa. Table 1 shows a summary of the 23 elements of the AGREE domains that were utilised to score the documents’ quality. Additionally, qualitative document analysis was used for systematic scrutiny; this method involved the examining and interpreting of a document’s content in order to draw its meaning and implications to SAM examination and care before, during and after a referral process [16, 17]. Therefore, we valued the identification of information pertinent to SAM referral process (Fig. 1) from information meant for other ailments or care outside the referral steps [17].

**Table 1:** Summary of AGREE II domains

AGREE domain	Summary of item questions
1. Scope and purpose	3 items: specific description of objectives, coverage of health questions and description of target population
2. Stakeholder involvement	3 items: guidelines development team variety, whether views of target population were sought and definitionof target users
3. Rigour of development	8 items: use of systematic evidence search methods, criteria for selecting evidence, description of strengths and limitations of evidence, description of recommendations formulation methods and risks of recommendations, link between evidence and recommendation, external review, and procedure for updating guidelines
4. Clarity of presentation	3 items: were recommendations specific and unambiguous, with different options and easily identifiable
5. Applicability	4 items: provision of tool for practice, describe barriers and facilitators to practice, consider resource implications and present monitoring and audit criteria
6. Editorial independence	2 items: not influenced by views of funding bodies and declare competing interests of guidelines development members

### Data sources: inclusion/exclusion criteria

Critical appraisal of the documents involved guidelines that address the care and referral of children with SAM. Through the consolidation of subject matter according to expert recommendations (three child health lecturers, a dietitian, a clinical associate and a doctor) and internet searches (google and government websites), we identified four guidelines (three national and one global) that are used (with the global guidelines adapted for local context) in South Africa (Box 1).

**Box 1** List of guidelines

**Table:** 

1. EDL: Standard Treatment Guidelines and Essential Medicines List for South Africa: Hospital Level Paediatrics (2013) 2. IMCI: Integrated Management of Childhood Illnesses Chart Booklet (2014) 3. IMC-SAM: Integrated Management of Children with Acute Malnutrition in South Africa: Operational Guidelines (2015) 4. WHO-Steps: Guidelines for the Inpatient Treatment of Severely Malnourished Children (2003)
*Note:* The EDL is a standard guideline for hospitals, IMCI is the likely to be used at primary healthcare facilities, WHO-Steps shaped severe acute malnutrition (SAM) care in South Africa and IMC-SAM is the current national guideline specifically for SAM care

### Quality and content assessment

#### Analytical framework

Healthcare guidelines are reviewed and assessed using various tools that reveal their complexity and technical quality [18]. We utilised components the WHO referral and AGREE frameworks (Fig. 1).

The WHO framework examines five components, namely initiating facility, referral practicalities, receiving facility, supervision and capacity-building [19]. In specifying stages of referral process, we broke down the WHO referral components of initiating and receiving a referral into seven steps (Diagnosis, Decision to refer, Stabilisation before referral, Stabilisation during referral, Care at receiving SAM referral, Decision to refer back, Rehabilitation plan after referral). Since the steps are descriptive in nature, the AGREE framework was incorporated to examine the documents analytically. Table 1 summarises the AGREE framework for policy development and content quality [18].

Comprehensiveness of the referral steps was mainly examined using three of the six domains (scope and purpose, clarity of presentation, applicability). These domains are specific to the SAM referral process, while the remaining three assess the rigour of guideline development processes (Fig. 1).

#### Document review process: AGREE and interviews

The guidelines (n = 4) were appraised by three individuals (except one, which was reviewed by two individuals), with two authors (FNM-C and EE) individually appraising all documents using an online portal and a further three individuals (doctor, dietitian and clinical associates) appraising one document each using hard copies. Responses were scored on a scale of 1 (strongly disagree) to 7 (strongly agree) on each of the AGREE tool domains.

To describe the referral representation and complement AGREE, five in-depth interviews were conducted with two academics, two frontline health workers (doctor and clinical associate) and a government official involved in guideline development. One government official, one academic and one frontline health worker commented on the EDL, a second health worker commented on the IMCI, while a third academic mainly commented on the IMC-SAM and added comments to other guidelines. The interviews explored the clarity of referral steps contained in the guidelines, adherence to the AGREE criteria and whether they could be recommended for use in various levels of SAM referral. Specifically, we sought information on stakeholder involvement, rigour of development (including use of evidence) and editorial independence (funders’ influence, competing interests).

### Data analysis

The lead researcher tabulated raw scores from all appraisers and emailed them for crosschecking and reconsideration before aggregation. The AGREE online portal scaled the percentage from all appraisers; in addition, we used the AGREE consortium (Box 2) formula to incorporate off-line appraisals.

**Box 2** AGREE’s scaled domain score formulae

**Table:** 

$$ {\frac{\mathrm{Obtained}\kern0.17em \mathrm{score}\hbox{--} \mathrm{minimum}\kern0.17em \mathrm{possible}\kern0.17em \mathrm{score}}{\mathrm{maximum}\kern0.17em \mathrm{possible}\kern0.17em \mathrm{score}-\mathrm{minimum}\kern0.17em \mathrm{possible}\kern0.17em \mathrm{score}}}^{\ast }100=\mathrm{Scaled}\kern0.17em \mathrm{Domain}\kern0.17em \mathrm{Score} $$
• Obtained score (for domain with 3 items) = item 1 (sum of appraisers core) + item 2 (sum of appraisers core) + item 3 (sum of appraisers core) = ? • Maximum possible score = 7 (strongly agree) × n (items) × 3 (appraisers) = ? • Minimum possible score = 1 (strongly disagree) × n (items) × 3 (appraisers) = ?

Maximum possible score was a sum of all scores of the individual item by scaling the total as a percentage of the maximum possible score for that domain

### Rigour

Co-authors (JE and PD) were responsible for ensuring all appraisals were equally incorporated in the final commentary of the SAM documents relevance in SAM referral. To ensure representation of the assessors’ input, the preliminary findings from the critical appraisal were mailed to all participants for member checking [20].

## Results

This section is composed of results from the guideline document versus SAM referral steps assessment, AGREE appraisals and expert interviews. The results are outlined for each document (aim of guideline, referral steps representation and AGREE scores/narratives) with an overall summary of the documents’ comprehensiveness. We report the guideline documents’ results in order of the oldest to the latest.

### WHO-Steps (2003): Guidelines for the Inpatient Treatment of Severely Malnourished Children

#### Aim of the guideline

WHO-Steps aims to improve global inpatient treatment of severe malnutrition in 10 steps. It is expected to address the SAM care problems alongside a WHO manual for managing SAM and the WHO/UNICEF strategy of IMCI. In South Africa, the 10 steps are embedded in two national guidelines (in this study: EDL and IMC-SAM) for hospital use only.

#### Referral steps representation

The guideline thoroughly elaborates in-hospital diagnosis/care and rehabilitation processes by emphasising appropriate case management and follow-up care [21]. The guidelines are meant for the receiving facility (hospital).

#### AGREE domain scores and narrative

The guideline scored high on scope and purpose (89%), clarity (75%) and applicability (61%), and lower on stakeholder involvement (44%), rigour of development (27%) and editorial independence (17%).

*Scope and purpose:* the objectives and populations were clearly articulated. It is meant to provide practical help to persons responsible for medical and dietary management of SAM children. Despite its overall clarity, the guidelines’ objectives and problem statements were not easily traceable by section titles.

*Stakeholder involvement:* a 44% score acknowledges that only a few experts contributed to the guidelines. Relevant professional groups or the public were not mentioned as being involved in reducing the stakeholder involvement strength. The specific users of guidelines were not stated, but rather spaces for implementation were identified as hospitals, therapeutic feeding centres, emergency situations and in nutrition rehabilitation centres [21]. It is unclear whether social workers and other professions in rehabilitation centres were obligated to implement the highly clinical recommendations.

*Rigour of development:* the procedures for updating the guideline and using evidence were not reported. Consideration of benefits, side effects and risks of recommendations were not fully discussed, but case–control-based recommendations, also in the 2013 update of malnutrition care, point to the use of research evidence. Additionally, WHO is known for use of systematic reviews and randomised trials [15].

*Clarity of presentation:* recommendations were easily identifiable by having a similar flow per step of care, containing various options of case management. Each step of SAM care started with conditions to note, how to treat, monitoring points, what to prevent and a reference for specific dosage and measures. However, WHO-Steps did not have in-text charts to guide diagnosis, classification of severity and treatment.

*Applicability:* it was made flexible for global dissemination, thus giving room to adaptation to nutritious ingredients and drug options that can be accessed in various contexts. However, the guideline was not thorough on facilitators or barriers of its application and, therefore, implications for resource-limited settings were unknown. Hence, applicability to context-specific conditions could not be guaranteed. It should be noted that the 10 steps have been updated per current evidence; thus the revised version and South Africa’s adapted version are respectively found in the 2013 WHO updates on SAM management and the 2015 South Africa’s operational guidelines for managing acute malnutrition [3, 4].

*Editorial independence:* the influence of funding bodies and competing interests on guideline development and/or group members were barely traceable. While disclosure of funders was disclosed, competing interests were unreported and thus unknown.

### EDL (2013): Standard Treatment Guideline and Essential Medicine List for South Africa – Hospital Level Paediatrics

#### Aim of the guideline

The EDL is a clinicians’ guide for quality, equitable and efficient healthcare in order to save the lives of babies and children [22]. It is meant for hospital-level paediatricians covering a wide range of diseases, including SAM.

#### Referral steps representation

EDL contains a comprehensive medicine list covering in-hospital level SAM diagnosis and treatment, decision to refer back and rehabilitation. However, the role of community health workers during rehabilitation was vague despite their importance in South Africa’s PHC strengthening.

#### AGREE domain scores and narrative

The guideline’s highest score were in scope (96%) and clarity (93%), followed by stakeholder involvement (63%), applicability (53%), rigour of development (44%) and editorial independence (9%). Table 2 contains supporting narratives.

**Table 2:** EDL (2013) AGREE domains support quotations

AGREE domains	Informants’ quotations
1. Scope and purpose	Quote 2a: “*In the EDL objectives are not clearly explained, for example, it is not stated how much percentage this intervention* [guideline] *is expected to reduce mortality from malnutrition*” (Informant 4, Paediatric Ward Doctor)
2. Stakeholder involvement	Quote 2b: “*The national essential drug list committee appointed by the Minister of Health* [national official] *includes health professionals, officials from the ministry of health and heads of pharmaceutical services from the provinces*” (Informant 2, Government Official) Quote 2c: “*They should involve clinical associates when developing these guidelines; we are in the field, we are likely to say what is needed*” (Informant 6, Clinical Associate)
3. Rigour of development	Quote 2d: “*Honestly, there is not always evidence thus sometimes only expert opinions are used. Particularly when new medicine has to be added then there has to be a systematic review of evidence and cost effectiveness of introducing a new medicine*” (Informant 2, Government Official) Quote 2e: “*In those forums the loudest voice wins, I wrote them to revise treatment per the new WHO recommendations, guess what, they overlooked my suggestion*” (Informant 3, Academic)
4. Clarity of presentation	Quote 2f: “*The EDL is very good for individuals experienced in SAM care, if not, some treatments are skipped since one has to go back and forth the pages. It could be best if the treatments were outlined in specific steps of execution for beginners or clinicians with general training*” (Informant 4, Paediatric doctor)
5. Applicability	Quote 2 g: “*affordability is taken into account at national level thus once something is in the EDL the affordable drugs team makes sure supplies are procured and made available. That’s the theory to say once something is in the EDL it should be available in the whole country*” (Informant 2, Government Official)
6. Editorial independence	Quote 2 h: “*The EDL is funded by national department of health and there is a process for managing conflict of interest. So no funding will ever be accepted from any supplier that has interest*” (Informant 2, Government Official)

*Scope and purpose:* EDL had a clear objective “*to reduce child mortality through offering quality healthcare*”, emphasising essential medicines, clearly identified target population (children) and maintained consistency with South Africa’s PHC guidelines [3]. However, EDL was limited in expressing outcome goals (quote 2a) and health questions were vaguely embedded in introductory sections.

*Stakeholder involvement:* based on acknowledgements, various teams were involved in EDL development. Singled out were various paediatric and national departments of health, including HIV/AIDS and child health clusters and, in particular, IMCI, tuberculosis and nutrition directories [22]. Despite appearing inclusive, team selection was political (quote 2b). Some relevant individuals were excluded. Individuals with less expertise but more conversant with remote area issues were side-lined (quote 2c). Additionally, ‘clinicians’ is used to identify target users but is unspecific.

*Rigour of development:* the methods of formulating recommendations and involving external reviewers and other health professions through emails were reported. However, there was no reporting of evidence search, selection and quality. The development process was mostly expert-based with use of some systematically searched evidence (quote 2d). Even where other experts contributed, inclusion of their inputs into the guidelines could not be guaranteed (quote 2e). Despite this, the guidelines had an updating procedure every 3 years, but its pace was slow since the current version is the third since 1998.

*Clarity of presentation:* its structure was clear with the segregation of diseases by chapters. The SAM chapter had headings for each treatment step sub-divided into prevention, detection, treatment and warnings. Different medication options were available; however, continuous reference to other chapters seemed unfriendly for a new user (quote 2f). Despite the shortfalls, key recommendations were easily identifiable and unambiguous.

*Applicability:* barriers and potential resource implications during implementation were not reported. One of the implementation barriers lay in accessing a mineral-vitamin mix ingredient for preparing the F75 and F100 feeds for stabilisation and catch-up (quote 4d under IMC-SAM). Nevertheless, a government official implied resource implications were taken into consideration (quote 2 g). EDL also had useful support tools, including feed recipes, tables and growth charts in appendices.

*Editorial independence:* the roles of EDL funders (the government) were not disclosed. Funding suppliers, such as pharmaceutical companies, were avoided (quote 2 h). Therefore, immunity from external influence was implied but disagreements among development teams were not reported.

### IMCI (2014) – Integrated Management of Child Illnesses

#### Aim of the guideline

South Africa adopted IMCI in 1997, originally developed by WHO/UNICEF in the 1990s. IMCI is meant to address serious challenges pertaining to provision of quality child healthcare by focusing on the wellbeing of the whole child [23]. Malnutrition is one of the major conditions included in IMCI.

#### Referral steps representation

The IMCI guided SAM examination, classification for decision to refer, and stabilisation before and during a transfer; however, the transit instructions were caregiver-centred. The IMCI was clearly for PHC and transit level guidelines.

#### AGREE domain scores and narrative

The IMCI scored highly on clarity (87%) and purpose (67%), but low on applicability (38%), rigour of development (24%), stakeholder involvement (17%) and editorial independence (17%). Despite covering PHC and transit recommendations, the IMCI scored the least because its development/adaptation process in South Africa was not reported. Table 3 contains supporting narratives.

**Table 3:** IMCI (2014) AGREE domains support quotations

AGREE domains	Informants’ quotations
2. Stakeholder involvement	Quote 3a: “*I think EMRS personnel* [paramedics in the ambulance] *are not targeted in this guideline and yet they are involved in most emergencies of SAM care*” (Informant 5, Clinical Associate)
5. Applicability	Quote 3b: “*…SA IMCI is adopted from WHO, however it includes HIV components not highly represented in the original IMCI by WHO*” (Informant 1, Academic)

*Scope and purpose:* the South Africa version of IMCI lacks an introductory section; thus, its scope and purpose are implicit, but the original version aims to reduce death, illness and disability, and to promote improved health among under-fives [23]. Its strength lays in the scope of diseases it covers and clarity in target population with sections specific to children 0 to 2 months and those from 2 months to 5 years.

*Stakeholder involvement:* we assumed South Africa IMCI abides by WHO requirements which state that all IMCI content should be carefully reviewed and revised (if needed) to make them consistent with the nationally adapted IMCI guidelines [23]. The IMCI handbook also identified doctors, nurses and other professions as users; however, users in the South Africa context remain vague with ambulance personnel not included in guideline dissemination (quote 3a). The low score on stakeholders also resulted from the absence of the list of developers, including information on whether public/patient opinions were sought.

*Rigour of development:* procedures for updating the guidelines were not reported. We assumed use of evidence due to guideline sections on clinical warnings about some treatments. Since the IMCI development process could not be easily traced, there was no guarantee about whether the evidence search processes, selection and their limits were rigorously engaged.

*Clarity of presentation:* well-articulated recommendations were arranged in the form of examination (by looking and tests), classification (colour-coded severity) and treatment. Recommendations were also specific and unambiguous with different options for stabilisation and referrals suggested for severe cases.

*Applicability:* South Africa-specific HIV/AIDS needs and evidence of implementation were included (quote 3b). Although not clear in the booklet, IMCI guidelines were adapted to include identification and management of HIV-infected and -exposed children. However, it is unknown whether the SAM treatments are feasible in resource-constrained and rural PHC facilities. Auditing criteria were also absent.

*Editorial independence:* with no report on competing interests and funders, we assumed the South African government was part of the IMCI development/adaptation process funding body. However, it was unknown whether the adaptation team was influenced by the government or WHO/UNICEF (as original developers).

### IMC-SAM (2015): Integrated Management of Children with Acute Malnutrition in South Africa – Operational Guidelines

#### Aim of the guideline

The IMC-SAM is the latest (2015) and only national guideline document entirely dedicated to acute malnutrition in South Africa. The IMC-SAM supplements a two-page national protocol for managing children with SAM (appendix 11 of guideline), which was developed in 2012 [3].

#### Referral steps representation

The IMC-SAM covered five steps of the SAM referral process, namely SAM examination, classification, diagnosis/care at hospital, decision to refer back and rehabilitation. IMC-SAM guided hospital and PHC facility practices but had minimal transit instructions. Additionally, IMC-SAM did not strongly stress stabilisation of SAM at PHC level.

#### AGREE domain scores and narrative

This document scored the highest of all the appraised documents with both scope and clarity scoring 89%, 74% on stakeholder involvement, 61% on applicability, 54% on rigour and 20% on editorial independence. The guideline followed the format of recent WHO documents and thus had more information on reasons for its development and its objectives. Table 4 contains supporting narratives.

**Table 4:** IMC-SAM (2015) AGREE domains support quotations

AGREE domains	Informants’ quotations
2. Stakeholder involvement	Quote 4a: “*When we did work in the Eastern Cape we did not seek public opinion, I don’t know whether before the national guidelines were developed whether they did* [involve the public]” (Informant 1, Academic)
3. Rigour of development	Quote 4b: “*Under the deputy director of the ministry of health, the development of a national guideline was spearheaded with the content highly dependent on WHO guidelines developed in 2013… its content as well as the IMCI are South Africa-specific by modifying the antibiotic step due to high prevalence of HIV*” (Informant 1, Academic)
5. Applicability	Quote 4c: “*With step 10* [of SAM care] *on rehabilitation we followed a sub-sample of discharged and found most of them had not gained much weight because they had limited amount and range of food, were not receiving enough money and were not accessing child grants which have just been introduced. On the basis of that, we did a lot of advocacy work, including a TV programme; this had a very big impact nationally which caused the minister to visit the next day and there were stuff in the Sunday Times* [local newspaper]*. That influenced the inclusion of* [a slogan saying] *‘make sure all children with SAM in the hospital are linked to social services’ in South Africa’s version of 10 steps of SAM care*” (Informant 1: Academic). Quote 4d: “*The ingredients were not mostly stored by facilities but the main problem was the mineral micronutrient mix because it did not appear in the EDL thus not accessible through normal channels thus it has to be made up so we found a friendly pharmacist in East London who made it up for the whole province* [pilot site-Eastern Cape Province]*, however he could not keep up with the demand*” (Informant 1, Academic)
6. Editorial independence	Quote 4e: “*There was a dispute from certain doctors on whether antibiotic should be given routinely (ampicillin). For F75 some doctors pushed very strongly for infant formulas since they were easier to get than rely on hospitals to make feeds; because of the issue of not binding on iron they cannot absorb thus likely to cause septicaemia and since F75 has no iron present it was endorsed around 2007*” (Informant 1, Academic)

*Scope and purpose:* its objectives were clearly stated; the questions covered by the guideline were stated under sub-headings. It was exceptional since it had ample information on the problem of SAM, ideal SAM care and its goals.

*Stakeholder involvement:* the guidelines clearly acknowledged developers and had well-identified target users. Contributors/developers were the nutrition and child directorate, child health and nutrition experts, international partners and members of the EDL committee. However, it is unknown whether public opinions were sought (quote 4a). Users of the guidelines were identified as doctors, nurses, dietitians and other healthcare workers responsible for the medical, dietary, social and rehabilitative management of SAM [3].

*Rigour of development:* the guidelines were informed by the 2013 evidence-based SAM care recommendations made in the WHO guidelines update document and the 2008 *Lancet* series on maternal and child undernutrition [3, 4]. Concurrently, other recommendations were adopted from the outcomes of the 10 steps of SAM care pilot study in Eastern Cape Province (quote 4b). The recommendations were therefore based on systematically searched, selected and well-evaluated evidence with acknowledged limitations [4]. However, the procedure for updating the guidelines was not explicit and use of South Africa-based pilot studies, aside from the Eastern Cape study, was not emphasised.

*Clarity of presentation:* it had specific, easily identifiable and unambiguous recommendations for both diagnosis and treatment of SAM with a diagram guiding decisions to refer at PHC level and treatment routes at hospital level. However, ease of use remained unsatisfactory since there was too much information for easy identification and follow through during implementation at different levels of care.

*Applicability:* Prior to IMC-SAM, there were strong piloting strategies of advocating for grants for all children with SAM and ensuring availability of resources for managing SAM in the Eastern Cape Province pilot site (quote 4c and 4d). The funds for the pilot were provided by those that contributed to the nationwide roll-out of 10 steps. Further, it is unknown whether all recommendations are applicable in resource-limited areas (quote 4d).

*Editorial independence:* publication by the government, support for training and advocacy from other organisations were disclosed. However, it was not transparent whether the organisations influenced the development procedures. Although the conflicts of the guideline development team were not disclosed, the 10 steps of SAM care adaptation revealed some conflicts. Among SAM pioneers, a prescription and care practice debate erupted and was resolved using evidence from research (quote 4e). Yet, the editorial independence seems weak because the influence of funders and competing interests of the IMC-SAM development team were not fully disclosed.

### Summary

**Fig. 2:**
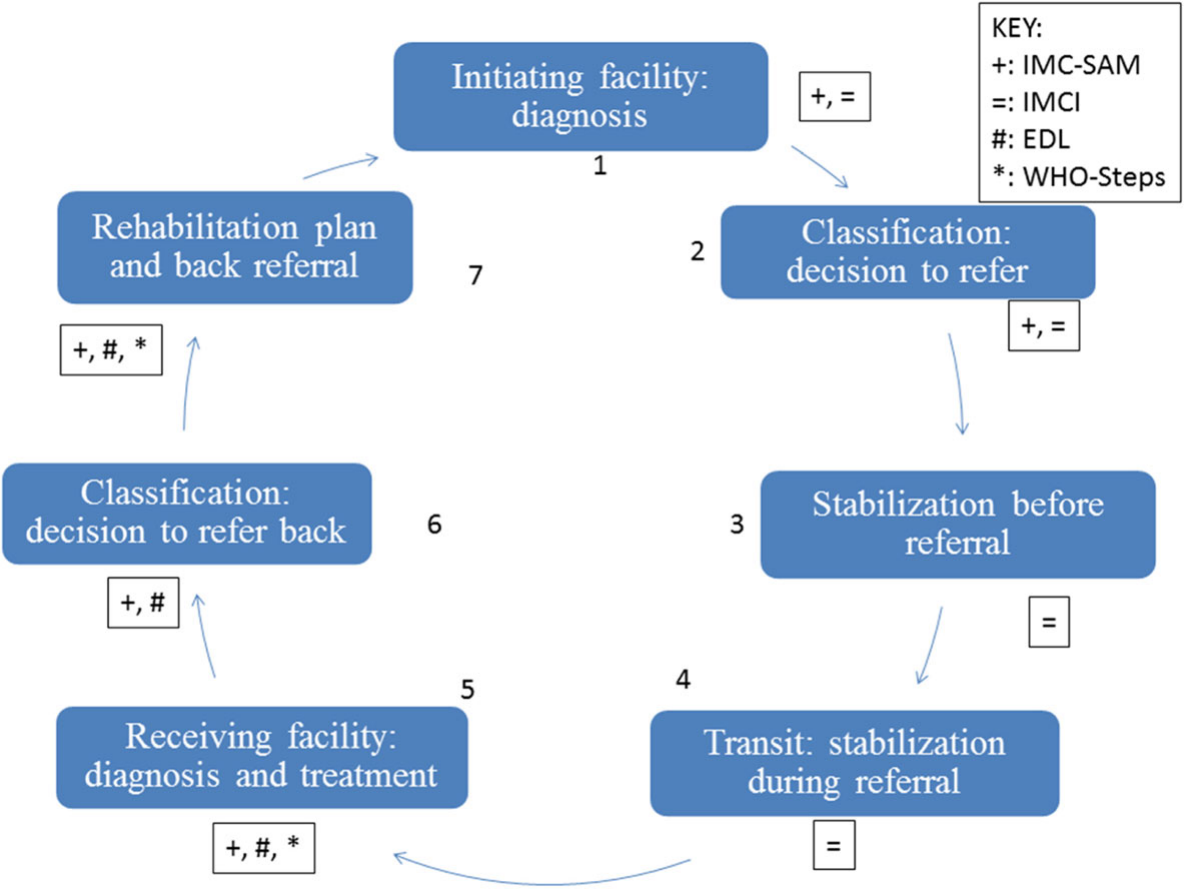
Guideline document recommendations to referral steps representation. + IMC-SAM: Integrated Management of Children with Acute Malnutrition in South Africa: Operational Guidelines (2015). = IMCI: Integrated Management of Childhood Illnesses Chart Booklet (2014). # EDL: Standard Treatment Guidelines and Essential Medicines List for South Africa: Hospital Level Paediatrics (2013). * WHO-Steps: Guidelines for the Inpatient Treatment of Severely Malnourished Children (2003)

All guidelines adequately addressed the seven referral practices. However, transit guidelines are vague since they are only in the IMCI, highly disseminated to facility-based healthcare practitioners and seldom disseminated to paramedics. Figure 2 depicts document versus referral step representation.

With guidelines disseminated to a variety of users (especially EDL and IMC-SAM), little demarcation was made based on qualifications of care providers. For instance, nurses cannot administer some antibiotics without a doctor’s instruction; therefore, it is unclear as to which parts of the guidelines are relevant to a nurse, doctor or dietitian.

Overall, all guidelines could be recommended for use with some modification on the stakeholders’ involvement, rigour of development, applicability and editorial independence. The guidelines appraised fell short on monitoring and auditing criteria for implementation such as process, behavioural and health outcome measures, which can shape quality of SAM care provision. A well formulated guideline uses rigorous methods, involves relevant stakeholders, including the public/patients, and discloses funders and their roles as well as competing interests.

## Discussion

Overall, all guidelines are recommendable for use at different levels of the SAM referral process. Guidelines must adhere to rigorous development processes, involvement of relevant stakeholders and consider implementation applicability. There is a need to address duplication across two national guideline documents (IMC-SAM and EDL) that currently give similar in-hospital care recommendations. Although presented differently with IMC-SAM incorporating recent methods and the EDL being intensive on drug options, there is a need to move to a standard best practice. However, for specific domains of AGREE, the documents had their own best and poorer practices.

*Scope, purpose and clarity of presentation*: The guidelines have clear objectives and recommendations but lack detail about which profession is responsible for specific guideline contents. Improvement of guidelines by identifying this is apt, especially in hospital settings where a child with SAM is under the care of doctors, nurses, dietitians and social workers.

*Rigour of development:* The IMC-SAM is the most recent (2015) and overall highest scoring amongst guidelines reviewed because it is disease specific, utilised recent evidence and was modelled over the recent WHO guidelines using the AGREE criteria [15]. However, all guidelines need to rate the quality of evidence used, especially for practice recommendations [4, 24]. Only IMC-SAM portrayed its use of various sources of evidence but not its strength. Clinical practitioners should be given an opportunity to judge the validity of recommendations; thus, a brief on how evidence is searched, selected and used is a necessity to enhance their dependability. It is therefore appropriate to incorporate the evidence-based approach, which integrates clinical expertise (education, experience and skills) and patient values to decide on ideal patient care [25].

Additionally, the absence of guideline update times is worrying since it leaves no assurance on whether new evidence is being incorporated. For instance, there is a 7-year gap between hospital level EDL revisions. The question on how long it should take for a guideline document to be reviewed remains unclear, with little or no debate.

*Stakeholder involvement:* determining which groups and individuals were relevant in the development process was problematic for all guidelines. To increase endorsement of guidelines, end-users must be incorporated [26]. Some stakeholders were side-lined, although the role of patients and their guardians is increasingly recognised [15]. Patients are vital because they are likely to prioritise the risks and benefits of treatment [27]. To enhance guideline applicability and patient satisfaction, future guideline development must include end-users and patients, now a WHO practice [15, 28].

*Applicability:* The potential of clinical practice guidelines to improve care and outcomes relies on their quality and independence [29]. In this case, quality includes consideration of resource implications and acknowledging potential funder influence. Since the South Africa guidelines are mostly modelled on WHO, their quality needs to be ensured by use of pilot studies. It is ideal practice to pilot test and overcome implementation barriers before guidelines are disseminated [24]. The piloting of the WHO 10 steps of SAM care in Eastern Cape Province is a good example, leading to social grants for the SAM rehabilitation phase to address poverty constraints and adapting antibiotics to suit HIV/AIDS care-related complications.

*Editorial independence:* a full disclosure of sources of funding, editorial independence from funders and conflicts among the guideline development teams is necessary in ensuring guideline independence from external forces [24]. Even in cases where funders influenced guideline contents and conflicts of interest are inevitable, developers are mandated to give valid justification for influence.

*AGREE process:* This study followed the procedures of AGREE. Potential bias is reduced by collating the views of several experts. However, we acknowledge a bias towards IMCI and WHO-Steps since there were no views from the developing teams; thus, our critiques are based on outsiders views. Furthermore, as the paper shows, utilising the commentaries of these and other experts provides insight into the meanings of the scores.

## Conclusion

In conclusion, the guidelines have clear scope and purpose for SAM referral healthcare. The clarity in presentation of care recommendations inform PHC and hospital level practices in the South Africa health system, but is inadequate to inform transit practices. Appraisals have revealed shortcomings in the guidelines development process and some gaps in implementation strategies. However, many SAM guidelines implementation difficulties may not be a result of poor clinical recommendations but of the health systems administrative challenges. These require research so that evidence can aid future guideline development. In the meantime, the IMC-SAM seems the best guideline since it has recent and more comprehensive recommendations.

## Abbreviations

AGREE: Appraisal of Guidelines for Research and Evaluation
EDL: Standard Treatment Guidelines and Essential Medicines List for South Africa: Hospital Level Paediatrics (2013)
IMCI: Integrated Management of Childhood Illnesses
IMC-SAM: Integrated Management of Children with Acute Malnutrition in South Africa: Operational Guidelines (2015)
PHC: primary healthcare
SAM: severe acute malnutrition
WHO-Steps: Guidelines for the Inpatient Treatment of Severely Malnourished Children (2003).
